# People with severe mental illness are not adequately screened for non-communicable diseases: Findings of a multi-country cross-sectional study in South Asia

**DOI:** 10.1192/j.eurpsy.2025.789

**Published:** 2025-08-26

**Authors:** K. K. Appuhamy, F. Wiggins, A. Mitchell, H. U. Ahmed, M. Ashworth, F. Aslam, J. R. Boehnke, O. P. Garcia, R. I. Holt, R. Huque, K. P. Muliyala, P. Murthy, B. Perry, D. Shiers, N. Siddiqi, K. Siddiqi, A. Tamizuddin, G. A. Zavala

**Affiliations:** 1 Health Sciences, University of York, York, United Kingdom; 2 National Institute of Mental Health, Dhaka, Bangladesh; 3 School of Life Course and Population Sciences, King’s College London, London, United Kingdom; 4 Institute of Psychiatry, Rawalpindi Medical University, Rawalpindi, Pakistan; 5 School of Health Sciences, University of Dundee, Dundee, United Kingdom; 6 Facultad de Ciencias Naturales, Universidad Autonoma de Queretaro, Santiago de Querétaro, Mexico; 7 Human Development and Health, Faculty of Medicine, University of Southampton, Southampton, United Kingdom; 8 ARK Foundation, Dhaka, Bangladesh; 9 National Institute of Mental Health and Neurosciences, Bangalore, India; 10 School of Psychology, University of Birmingham, Birmingham; 11 Psychosis Research Unit, Greater Manchester Mental Health NHS Trust, Manchester, United Kingdom

## Abstract

**Introduction:**

People with severe mental illness die 10-20 years earlier than the general population. This is largely due to non-communicable diseases (NCDs) such as hypertension, diabetes and hypercholesterolaemia increasing the risk of cardiovascular disease, which is the greatest contributor to the excess mortality seen. The effect of these NCDs is likely to be greater in low-and middle-income countries such as Bangladesh, India and Pakistan due to additional barriers to health care access, lack of resources and other sociodemographic variables.

**Objectives:**

Our study aimed to estimate the proportion of individuals with SMI in Bangladesh, India, and Pakistan who were screened for NCDs and offered health risk modification advice. Furthermore, we also explored socio-demographic factors associated with the likelihood of being screened for NCDs within this demographic.

**Methods:**

This cross-sectional study gathered data from three national mental health institutions in South Asia. Participants aged ≥18 years diagnosed with SMI were included. Data collection involved face-to-face interviews based on the World Health Organisation Stepwise (WHO-STEPS) approach to NCD risk factor surveillance, supplemented by anthropometric measurements and blood tests to confirm NCDs. The prevalence of screening, diagnosis, health risk modification advice, and treatment for diabetes, hypertension, and high cholesterol was assessed. A logistic regression model assessed the associations of sociodemographic characteristics with NCD screening.

**Results:**

3,989 participants were recruited. Screening prevalence varied by country and disease, with hypertension being the most commonly screened NCD (Bangladesh = 52.5% [50.0-55.1], India = 43.1% [40.3-45.9], Pakistan = 60.9% [58.2-63.5]), and cholesterol was the least common (Bangladesh = 4.1% [3.2-5.2], India = 14.8% [12.9-17.0], Pakistan = 9.6% [8.1-11.3]). Characteristics such as BMI, age and education level were positively associated with screening, and females were more likely to be screened than males. The provision of health risk modification advice was most common in India (diet = 66.7% [62.1-71.1], physical activity = 71.5% [67.0-75.6], smoking = 17.1% [13.8-21.0]), and least common in Bangladesh (diet = 17.8% [15.8-20.0], physical activity = 12.0% [10.3-13.8], smoking = 9.8% [8.3-11.5]).

**Conclusions:**

There is a consistent gap in the screening of NCDs among individuals with SMI in South Asia, with marked sociodemographic disparities. There is a pressing need for standardised screening protocols and health risk modification interventions tailored to South Asian populations. Improving health literacy and implementing culturally sensitive, cost-effective prevention strategies could mitigate the increased risk of NCDs in South Asian individuals with SMI.

**Disclosure of Interest:**

None Declared

